# Epidemiology of TB in prisoners: a metanalysis of the prevalence of active and latent TB

**DOI:** 10.1186/s12879-022-07961-8

**Published:** 2023-01-11

**Authors:** Aline Ferreira Placeres, Débora de Almeida Soares, Felipe Mendes Delpino, Heriederson Sávio Dias Moura, Alessandro Rolim Scholze, Márcio Souza dos Santos, Ricardo Alexandre Arcêncio, Inês Fronteira

**Affiliations:** 1 Instituto de Higiene e Medicina Tropical (IHMT), Universidade Nova de Lisboa, Rua da Junqueira 100, 1349-008 Lisbon, Portugal; 2 Programa de Pós Graduação em Enfermagem, Universidade Federal de Pelotas, Pelotas, Brazil; 3 Escola de Enfermagem de Ribeirão Preto (EERP/USP), Universidade de São Paulo, São Paulo, Brazil; 4 Universidade Estadual do Norte do Paraná, Campus Luiz Meneguel de Bandeirantes, Bandeirantes, Brazil; 5 Global Health and Tropical Medicine, Instituto de Higiene e Medicina Tropical, Universidade NOVA de Lisboa, Lisbon, Portugal

**Keywords:** Meta-analysis, Prevalence, Tuberculosis, Prisoners, Prisons, Population deprived of liberty

## Abstract

**Background:**

Tuberculosis (TB) in prisons usually occurs at higher rates than in the general population, especially in developing countries. TB has been reported as the most common cause of death among prisoners. Studies have shown limitations for early detection of TB in prisons that seem to result from mistaken concepts about TB, delayed diagnosis mainly due to the naturalization of lack of healthcare for this population

**Methods:**

A scoping review was performed using the methodology of the Joanna Briggs Institute to assess “What are the scientific evidences on the epidemiology of TB in the prison system?”. Then, a meta-analysis was performed to assess the prevalence of TB (active and latent) TB in prisoners. The results are presented as prevalence, in percentage, through random effects models, with a confidence interval of 95%.

**Results:**

Regarding active TB, the results of the metanalysis showed that countries with a high burden of TB had a prevalence of 3.54% [2.71; 4.63], countries not considered to be high burden TB countries had a prevalence of 1.43% [0.86; 2.37]. Latent TB had a prevalence of 51.61% [39.46; 63.58] in high TB burden countries and a prevalence of 40.24% [23.51; 59.61] in countries with low TB burden. In terms of development, in low- and lower-middle-income countries, the prevalence of active TB was 3.13% [1.84; 5.29] and in high- and upper-middle income countries the prevalence was 2.25% [1.70; 2.99]. The prevalence of latent TB in high- and middle-income countries was 43.77% [28.61; 60.18] and of 49.42% [45.91; 52.94] in low and lower middle-income countries.

**Conclusion:**

Our analysis suggests that TB, and probably other infectious diseases, find fertile ground in prisons where previous acquire social disadvantages seem to thrive—therefore, TB in prisons is a global public health problem and effective strategies are needed to control the disease are needed targeting the prison environment, including rapid health assessments to understand each context and to implement tailored and precision interventions.

**Supplementary Information:**

The online version contains supplementary material available at 10.1186/s12879-022-07961-8.

## Background

Tuberculosis (TB) is a communicable disease and one of the ten leading causes of death worldwide. It is also the leading cause of death by a single infectious agent, surpassing HIV [1].

TB in prisons usually occurs at higher rates than in the general population, especially in developing countries. TB has been reported as the most common cause of death among prisoners [2]. A disproportionated number of prisoners belong to population groups that already present a high risk of infection and TB disease (e.g., substance users, homeless individuals, individuals with mental illness, ex-convicts, HIV/AIDS and immigrants) and have often higher prevalence of TB [3, 4].

During incarceration, prisoners face living conditions, such as crowding, absent or precarious basic sanitation and housing infrastructure, malnutrition, poor ventilation, deficient lighting, illicit drug use and difficulty in accessing health services, that make the prison system a potential TB-transmitting (and aggravating) environment [2–5].

Studies have shown limitations for early detection of TB in prisons that seem to result from mistaken concepts about TB, delayed diagnosis mainly due to the naturalization of lack of healthcare for this population, interpretation of prisons as places of "death" and "suffering" and, in some situations, deprivation of the right to health for detainees due to their position before society [6, 7]. These factors translate into inequities in accessing health services, including TB related prevention, diagnosis, and/or treatment services. Studies also point out the misconceptions of the right to health of prisoners and consequently, the need to change the understanding of this basic human right [8].

TB in prisoners is, thus, a major public health concern that contributes to the dissemination of TB and the perpetuation of its effects not only in this population but in society in general.

Several types of reviews have addressed the issue of TB in prisoners (12 review studies, 6 systematic reviews, 2 metanalysis, 2 integrative reviews, 1 comprehensive review and 1 scoping review) [8–19]. Nevertheless, none comprehensively addressed scientific evidence of the epidemiology of TB in the prison system worldwide.

We conducted a scoping review (SR) to address the review question “What are the scientific evidences on the epidemiology of TB in the prison system?”. For the purposes of this SR, we defined epidemiology as events, states, health processes and factors involving TB in prisoners described as incidence, prevalence, associations, comparisons, predictions, correlations, descriptions, risks, trends or probabilities resulting from studies.

The large number of prevalence results found led us to carry out the meta-analysis reported in this paper.

## Methods

A SR was performed using the methodology of the Joanna Briggs Institute [20].The protocol for this scoping review is registered in the Open Science Framework under https://osf.io/vn2cw/.

### Search strategy

Databases were searched in June 2021. No time limit was applied. We searched Medical Literature Analysis and Retrieval System Online (MEDLINE/PubMed)*;* Latin American and Caribbean Literature in Health Sciences (LILACS); Scopus (Elsevier) and Scientific Electronic Library Online (SciELO) using the following search terms and keywords: Tuberculosis, Kochs Disease, Koch's Disease, Koch Disease, Mycobacterium tuberculosis Infection, Mycobacterium tuberculosis Infections, Prisons, Prison, Penitentiaries, Penitentiary, Prisoners, Prisoner, Prison inmate. The search strategies used in each database are detailed in Additional file 1.

### Eligibility criteria

#### Inclusion criteria

We adopted the strategy: Population (P), Concept (C), Context (C) [21] as defined in Table 1.

**Table 1:** Data search strategy

Population	Persons deprived of liberty of both sexes, who are responsible for the violation of criminal law as an adult in the country where the study was conducted
Concept	Quantitative studies in prisons that address the prevalence of TB
Context	Cross-sectional, cohort studies, randomized clinical trials, case–control, ecological studies in Portuguese, English and Spanish with no time limit and which investigated the categories mentioned in Concept (C)

Studies that presented cases of TB in all clinical forms (i.e., all forms of active TB and latent TB, considered the inactive form of the disease) from A-15 to A-19 according to the International Classification of Diseases version 10—ICD-10 were included [22].

#### Exclusion criteria

We excluded publications that investigated other infectious diseases and that had no data on TB, that addressed the relationship between TB and drug use, studied immigrant population who were also deprived of liberty, TB coinfection with HIV and/or Hepatitis C; molecular epidemiology of TB, TB lineage variation; TB after leaving the prison system; drug resistant TB; sensitivity testing methods; performance of scores for TB screening; case studies; cost of TB tests in prisons; prisoners of war with TB; summaries of official TB control consensus documents in the prison system; and qualitative studies.

#### Selection process

References retrieved from databases were assessed independently (title and abstract) by two reviewers (AP and DS), after removal of duplicates. Disagreements were resolved by a third reviewer (IF). Publications eligible for full-text reading were again assessed independently by two reviewers (AP and DS).

#### Data extraction and analysis

Data extraction was conducted by AP and DS using an adapted version of the form of the JBI [20] model according to the specificities of this SR that included: type of study, language, aims, population, concept, context, source of evidence, citation details, country, methods and relevant results.

Included studies were divided into the following categories: prevalence, incidence, associated factors, treatment outcomes, environmental factors and effects of prevention programs. In this paper, we report only on included studies that presented prevalence data. All studies addressing prevalence of TB were assessed in terms of quality and risk of bias and subjected to meta-analysis.

### Assessment of quality and risk of bias

We used the JBI critical appraisal tools for prevalence studies and for cross-sectional studies [23]. Critical appraisal was conducted by DS, AS, MS and HM. AP was consulted whenever there were questions or doubts. The Checklist for Analytical Cross-Sectional Studies was used when the studies did not have as main objective to evaluate the prevalence of TB, but presented TB prevalence data. The Checklist for Prevalence Studies was used when the aim of the study was to evaluate the prevalence of TB.

Each checklist consists of eight (cross-sectional) or nine questions (prevalence), with four options of answer: yes; no; unclear and not applicable. To decide on the quality of the studies and to define which were low, medium or high quality we attributed a score to each item, being, 2 for yes or not applicable, 1 for unclear and 0 for no. The score obtained in each question was then added to reach a final score. For the critical appraisal tool for cross-sectional studies, low quality refers to studies scoring 0 to 8, medium quality to studies scoring 9 to 12 and high quality to studies scoring higher than 12. For the critical appraisal tool for prevalence studies, low quality corresponded to a 0 to 9 score, medium quality to a 10 to 14 score and high quality to a score of 15 or higher.

This study had the publication bias of not analyzing the gray literature, in addition to not including studies that were not published because they did not present data considered relevant for publication.

### Meta-analysis

Meta-analysis was performed to assess the prevalence of TB (active and/or latent) in prisoners. The results are presented as prevalence, in percentage, through random effects models, with a confidence interval of 95%. The heterogeneity between the studies was evaluated using the I2 test, in which values ​​above 50% and p-value less than 0.05 were considered as high heterogeneity [24].

To be included in the meta-analysis, the study had to inform on the number of people with TB, active and/or latent, and the total sample size. If the study only provided data on the percentual prevalence, the number of people was calculated. Analysis was stratified according to the burden of TB in the country where the study was conducted (high burden country for TB/ not high burden country for TB). We used the list of countries of high TB burden of the Global Tuberculosis Report of 2020 [25]. Analysis was also stratified by income level as defined by the World Bank (high- or upper-middle-income/ low- or lower-middle-income country) [26]. To analyze the trend in TB prevalence, we used the years of data collection for each study. Analysis was performed using the R programming language, using the Meta package [27].

## Results

### Characteristics of the studies

**Fig. 1:**
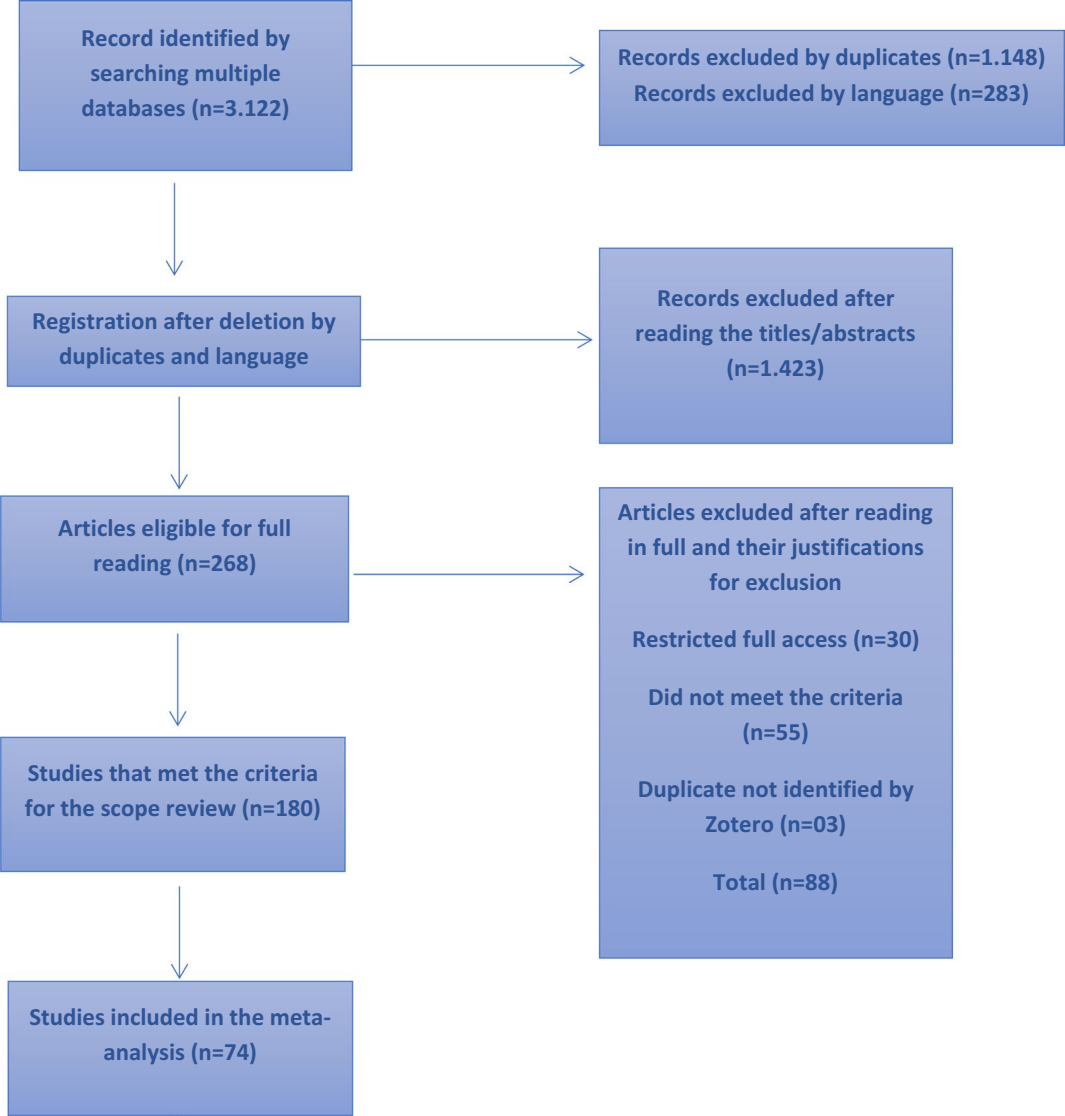
PRISMA flow diagram illustrating the search process which eligible articles was identified for data synthesis

Searches of the databases yield a total of 3122 articles (1300 from PubMed, 1643 from Scopus, 94 from LILACS and 85 from SciELO) of which 1148 were duplicates and 283 were in other languages besides those defined for this SR. A further 1493 articles were excluded after applying eligibility criteria to title and abstract. A total of 268 publications were considered for full-text assessment, of which 30 were excluded because it was not possible to access them, 55 did not meet the eligibility criteria (16 were qualitative studies, 8 addressed Multi-drug resistant TB (MDR-TB), 5 studied prisoners with co-infection TB-HIV, 3 compared strains of Koch bacilli, 3 concerned TB outbreaks, 2 studied homeless people, 2 were on-going clinical trials, 2 jointly addressed prison officials and prisoners, 3 were editorials, 3 were commentaries, 3 were on vulnerable populations as a whole, 2 were legislative reviews, 3 specific evaluations and tests on incarceration rate, increased risk of TB in the general population or juvenile prisoners) and 3 were duplicates not previously identified. A total of 180 articles met the inclusion for the initial scoping review and of these 74 studies presented prevalence results and were included in the meta-analysis reported in this paper (see Fig. 1).

Of the 74 studies with prevalence results, 59 included data on the prevalence of active TB and 15 on the prevalence of latent TB in prisons and were analysed separately. The country with the highest number of manuscripts was Brazil (n = 19), followed by Ethiopia (n = 9), USA (n = 5), Spain (n = 3) and Zambia (n = 3), Malaysia (n = 2), Malawi (n = 2), Pakistan (n = 2), Bangladesh (n = 2), South Africa (n = 2) and India (n = 2), Turkey (n = 2), Colombia (= 2) (Table 2). Several countries had only one study each. Publications dates ranged from 1986 to 2021. The number of participants ranged from 164 to 622,000 prisoners.

**Table 2:** Results of quality appraisal

Reference numbers	References	Points	Quality	Country
[43]	Santos AS, Oliveira RD, Lemos EF et al. Clin Infect Dis, 2021, 72(5): 771–777	17	High	Brazil
[44]	Al-Darraji HA, Altice FL, Kamarulzaman A. Trop Med Int Health, 2016, 21(8); 1049–1058	18	High	Malaysia
[45]	White MC, Tulsky JP, PortilloCJ. 2001, Int J Tuberc Lung Dis, 5(5):400–404	16	High	USA
[46]	Sretrirutchai S, Silapapojakul K, Palittapongarnpim P et al., 2002, Int J Tuberc Lung Dis, 6(3):208–214	15	High	Thailand
[47]	Nyangulu DS, Harries AD, Kang'ombe C et al. 1997, Lancet, 350(9087): 1284–1287	15	High	Malawi
[48]	Habeenzu C, Mitarai S, Lubasi D et al. 2007, Int J Tuberc Lung Dis, 11(11):1216–1220	15	High	Zambia
[49]	Aily DCG, Berra JA, Brandão AP, Chimara EP. 2013, Rev. Inst. Adolfo Lutz, 72(4): 288–294	16	High	Brazil
[50]	Macedo LR, Maciel EL, Struchiner CJ et al. 2017, Epidemiol. serv. Saúde, 26(4): 783–794	18	High	Brazil
[51]	Henostroza G, Topp SM, Hatwiinda S et al. 2013, 8(8): e67338	17	High	Zambia
[52]	Sanchez A, Larouzé B, Espinola AB et al., 2009, Int J Tuberc Lung Dis, 13(10): 1247–1252	18	High	Brazil
[53]	Noeske J, Kuaban C, Amougou G et al. 2006, East Afr Med J, 83(1): 25–30	18	High	Cameroon
[54]	Aerts A, Habouzit M, Mschiladze L et al.2000, Int J Tuberc Lung Dis, 4(12): 1104–1110	18	High	USA
[55]	Banu S, Hossain A, Uddin M et al. 2010, PLoS One, 5(5): e10759	18	High	Bangladesh
[56]	Alarcón-Robayo J, Martinez-Casallas L, Samir-Sánchez Met al. 2016,Acta méd. peru, 33(3): 202–207	17	High	Colombia
[57]	Lemos AC, Matos ED, Bittencourt C et al. 2009, Jornal Brasileiro de Pneumologia, 35(1):63–68	18	High	Brazil
[58]	Winetsky DE, Almukhamedov O, Pulatov D et al. 2014, PLoS One, 9(1): e86046	18	High	Republic Tajikistan
[59]	Valença MS, Scaini JLR, Abileira FS et al., 2015, Int J Tuberc Lung Dis 19(10): 1182–1187	18	High	Brazil
[60]	Berihun YA, Nguse T, Gebretekle G et al., 2018, Ethiop J Health Sci, 28(3): 347–354	18	High	Ethiopia
[61]	Kalonji G, De Connick G, Kazumba Nsaka D et al., 2016, Trop Med Health, 44:30	18	High	Democratic Republic of Congo
[62]	Addis AZ, Adem E, Alemu A et al. 2015, Asian Pac J Trop Med, 8(2): 127–131	18	High	Ethiopia
[63]	Fuge TG, Ayanto S. 2016, BMC Res Notes, 9:201	18	High	Ethiopia
[64]	Moges B, Amare B, Asfaw F et al. 2012, BMC Infect Dis, 12: 352	18	High	Ethiopia
[65]	Sanchez A, Gerhardt G, Natal S et al. 2005, Int J Tuberc Lung Dis, 9(6): 633–639	18	High	Brazil
[66]	Séri B, Koffi A, Danel C et al. 2017, PLoS One, 12(7): e0181995	16	High	Côte d’Ivoire
[67]	Ali S, Haileamlak A, Wieser A et al. 2015, PLoS One, 10(12): e0144040	16	High	Ethiopia
[68]	Owokuhaisa J, Thokerunga E, Bazira J et al. 2014, Adv Res, 2(11): 618–625	18	High	Uganda
[69]	Adesokan HK, Cadmus EO, Adeyemi WB et al. 2014, Afr J Med Med Sci, 43(Suppl 1): 45–50	18	High	Nigeria
[70]	Vieira AA, Ribeiro AS, Siqueira AM et al. 2010, Rev. bras. Epidemiol, 13(4): 641–650	18	High	Brazil
[71]	Jordan AM, Podewils LJ, Castro KG et al.2019, Int J Tuberc Lung Dis, 23(11): 1198–1204	18	High	South African
[72]	Gizachew Beza M, Hunegnaw E, Tiruneh M et al. 2017, Int J Bacteriol, 2017: 3,826,980	18	High	Ethiopia
[73]	Hatwiinda S, Topp S.M, Siyambango M et al. 2018, Trop Med Int Health, 23(2): 243–250	16	High	Zambia
[74]	Valença MS, Cezar-Vaz MR, Silva PD et al. 2016, Ciência & Saúde Coletiva, 21(7): 2111–2122	16	High	Brazil
[75]	Pedro HS, Nardi SM, Pereira MI et al. 2011, Rev. patol. Trop. 40(4): 287–295	13	High	Brazil
[76]	Gray BJ, Perrett SE, Gudgeon B et al. 2020, J Public Health (Oxf), 42(1): e12-e17	16	High	United Kingdom
[77]	Bhatnagar T, Ralte M, Ralte L et al. 2019, PLoS One. 14(7): e0219988	18	High	India
[78]	Katyal M, Leibowitz R, Venters H et al. 2018, J Correct Health Care, 24(2): 156–170	18	High	USA
[79]	Merid Y., et al. The International Journal of Tuberculosis and Lung Disease, Volume 22, Número 5, 1 de maio de 2018, pp. 524–529 (6)	16	High	Ethiopia
[80]	Telisinghe L. et al. January 2014. PLOS One. Volume 9. Issue 1, e87262	16	High	South Africa
[81]	Adane, K. et al. February, 2016. PLOS One. https://doi.org/10.1371/journal.pone.0149453	16	High	Ethiopia
[82]	Banu, S., et al. May, 2015. PLOS One	16	High	Banglades
[83]	Carbonara, S., et al. 2005, European Respiratory Journal. 25: 1070–1076; DOI: 10.1183 / 09,031,936.05.00098104	16	High	Italy
[84]	Cunha UA, Marques M, Evangelista MS et al. 2018, Rev. Soc. Bras. Med. Trop, 51(3): 324–330	18	High	Brazil
[85]	Sanchez A, Veronique M, Gerhardt G et al. BMC Public Health, 2013, 13: 983	14	High	Brazil
[86]	Kiter G, Arpaz S, Keskin S et al., 2003, Int J Tuberc Lung Dis, 7(2): 153–158	18	High	Turkey
[87]	Mandizvidza A, Dlodlo RA, Chinnakali P et al. 2020, Tuberc Res Treat: 5,829,471. https://doi.org/10.1155/2020/5829471	18	High	Zimbabwe
[88]	Koo DT, Baron RC, Rutherford GW. 1997, Am J Public Health, 87 (2):279–282	18	High	USA
[89]	Nogueira PA, Abrahão RMCM. 2009, Rev. Bras. Epidemiol, 12(1):1–8	14	High	Brazil
[90]	Aguilera XP, González C, Nájera-De Ferrari M et al. 2016, Int J Tuberc Lung Dis, 20(1):63–70	16	High	Chile
[91]	Adib SM, Al-Takash H, Al-Hajj C, 1999, Eur J Epidemiol, 15(3):253–260	18	High	Lebanon
[92]	Abrahão RMCM, Nogueira PA, Malucelli MIC, 2006, Int J Tuberc Lung Dis, 10(2):203–208	18	High	Brazil
[93]	Nogueira PA, Abrahão RM, Galesi VMN. 2012, Revista de Saúde Pública, 46 (1): 119–127	18	High	Brazil
[94]	Margolis B, Al-Darraji HAA, Kamarulzaman A et al., Int J Tuberc Lung Dis, 17(12):1538–1544	16	High	Malaysian
[95]	Chekesa B, Gumi B, Chanyalew M et al. 2010, PLoS One, 15(5): e0233314	18	High	Ethiopia
[96]	Navarro PD, Almeida IN, Kritski AL et al. 2016, Jornal Brasileiro de Pneumologia, 42(5): 348–355	18	High	Brazil
[97]	López de Goicoechea-Saiz ME, Sternberg F, Portilla-Sogorb J et al. 2018, Revista Española de Sanidad Penitenciaria, 20(1): 4–10	16	High	Spain
[98]	Rueda Z.V., et al.The International Journal of Tuberculosis and Lung Disease, Volume 18, Number 10, 1 October 2014, pp. 1166–1171(6)	14	High	Colômbia
[99]	Gárcia-Guerrero, J., et al. 2010. Revista espanhola de sanidade penitenciaria. vol.12 no.3	18	High	Spain
[100]	Ritter C, Elger BS. 2012, Int J Tuberc Lung Dis,16(1): 65–69	16	High	Switzerland
[30]	Anderson KM, Keith EP, Norsted SW, 1986. Chest, 89(6):817–821	12	Medium	USA
[31]	Kuhleis D, Ribeiro AW, Costa ERD, Cafrune PI et al. 2012, Memórias do Instituto Oswaldo Cruz,107(7):909–915	14	Medium	Brazil
[32]	Assefzadeh M, Barghi R, Shahidi S et al. 2009, East Mediterr Health J, 15(2): 258–263	14	Medium	Iran
[33]	Prasad BM, Thapa B, Chadha SS et al. 2017, Int J Infect Dis, 56: 117–121	12	Medium	India
[34]	Shah SSA, Ali M, Ahmad M et al. 2013, Pak. J. Med. Health Sci.7(1): 172–175	12	Medium	Pakistan
[35]	Öngen G, Börekçi S, İçmeli OS et al. 2013, Tuberk Toraks, 61(1): 21–27	14	Medium	Turkey
[36]	Chiang CY, Hsu PK, Suo J et al.2002, J Formos Med Assoc, 101(80): 537–541	13	Medium	Taiwan
[37]	Shrestha G, Yadav DK. Gautam R et al. 2019, Tuberc Res Treat: 3,176,167	14	Medium	Nepal
[38]	Salazar-De La Cuba et. al. Trppical and Medicine International Health. March 2019, Volume24, Issue3, Page: 328–338	12	Medium	Peru
[39]	Mallik, G., et al. March 2017, Public Health Action, Volume 7, Number 1, pp. 67–70(4)	10	Medium	India
[40]	Costa-Junior A, Silva Júnior JL, Costa AC et al. 2016, Rev. patol. Trop, 45(1):12–22	13	Medium	Brazil
[41]]	Martín V, Brugos M, Valcarcel I et al. 2000, Revista Española de Salud Pública, 74(4)	14	Medium	Spain
[42]	Hussain H, Akhtar S, Nanan D et al. 2003, Int J Epidemiol, 32(5): 794–799	14	Medium	Pakistan
[27]	Cerecer Callú P, Aranda Lozano JL, Márquez Fiol AR et al. 2006, Enferm. Infecc. Microbiol, 26(4):94–100	8	Low	Mexico
[28]	Kanyerere HS, Banda RP, Gausi et al. 2012, 2(1): 10–14	8	Low	Malawi
[29]	Reis AJ, David SM, Valim AR et al. 2016, J. bras. Pneumol, 42(4): 286–289	8	Low	Brazil

### Quality of evidence

Only three of 74 studies assessed had low quality [27–29], 13 had medium quality [30–42] and 58 were considered high quality [43–100].

The low quality studies evaluated the prevalence of TB in populations or samples of prisoners without TB symptoms [27, 28] and one study evaluated the prevalence only in symptomatic prisoners [29]. Three of the medium quality studies evaluated the prevalence of latent TB [40–42], nine TB in non-symptomatic patients [30–38] and one the prevalence of TB in symptomatic patients [39]. Results of quality appraisal are presented in Table 2.

### Meta-analysis

**Fig. 2:**
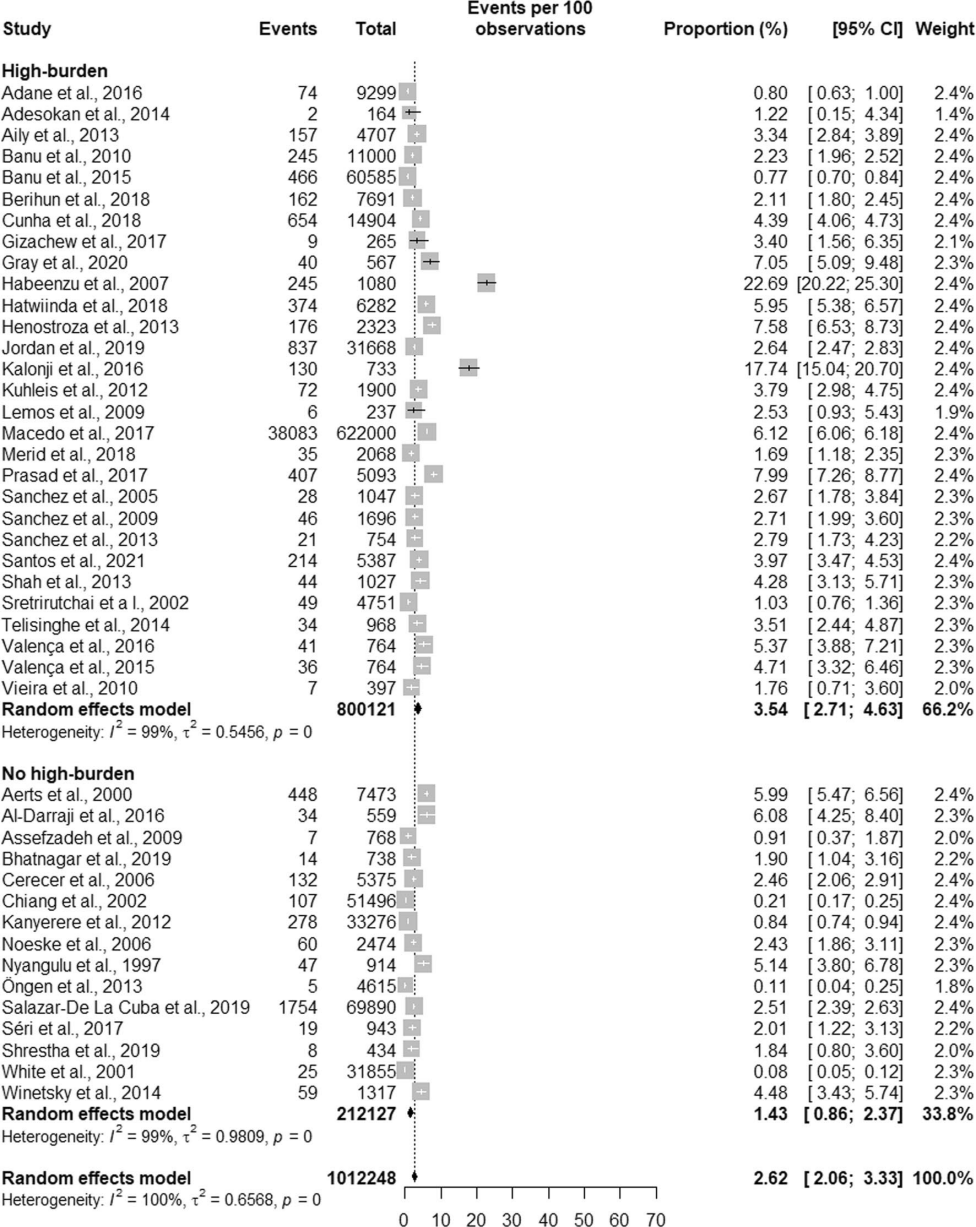
Prevalence of general tuberculosis by burden of TB of the country

Regarding active TB, the results of the metanalysis showed that countries with a high burden of TB had a prevalence of 3.54% [2.71; 4.63] with a total population in all studies of 800,121 prisoners. Countries not considered to be high burden TB countries had a prevalence of 1.43% [0.86; 2.37] with a total population in all studies of 212,127 prisoners. Still in relation to active TB, when analysing all countries together, the overall prevalence of active TB was 2.62% [2.06; 3.33] adding up to a total population of 1,012,448 prisoners (see Fig. 2).

**Fig. 3:**
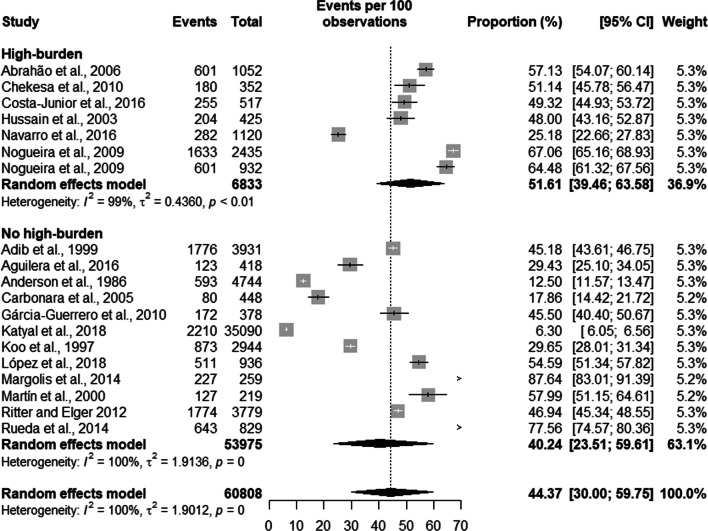
Prevalence of latent tuberculosis by burden of TB of the country

Latent TB had a prevalence of 51.61% [39.46; 63.58] with a population of 6,833 prisoners in high TB burden countries and a prevalence of 40.24% [23.51; 59.61] and a population of 53,975 prisoners in countries with low TB burden. The overall prevalence of latent TB was 44.37% [30.00; 59.75] with a total population of 60,808 prisoners (see Fig. 3).

**Fig. 4:**
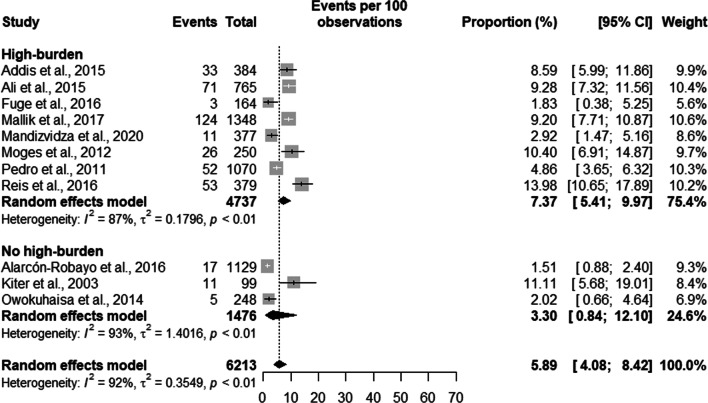
Prevalence of symptomatic tuberculosis by burden of TB of the country

Prisoners with suggestive TB symptoms had a prevalence of TB of 7.37% [5.41;9.97] in high burden countries with a total population of 4737 prisoners, and a prevalence of 3.30% [0.84;12.10] in a population of 1476 in low burden countries. Overall, the prevalence of TB in symptomatic prisoners was 5.89% [4.08;8.42] in a population of 6,213 prisoners (see Fig. 4).

**Fig. 5:**
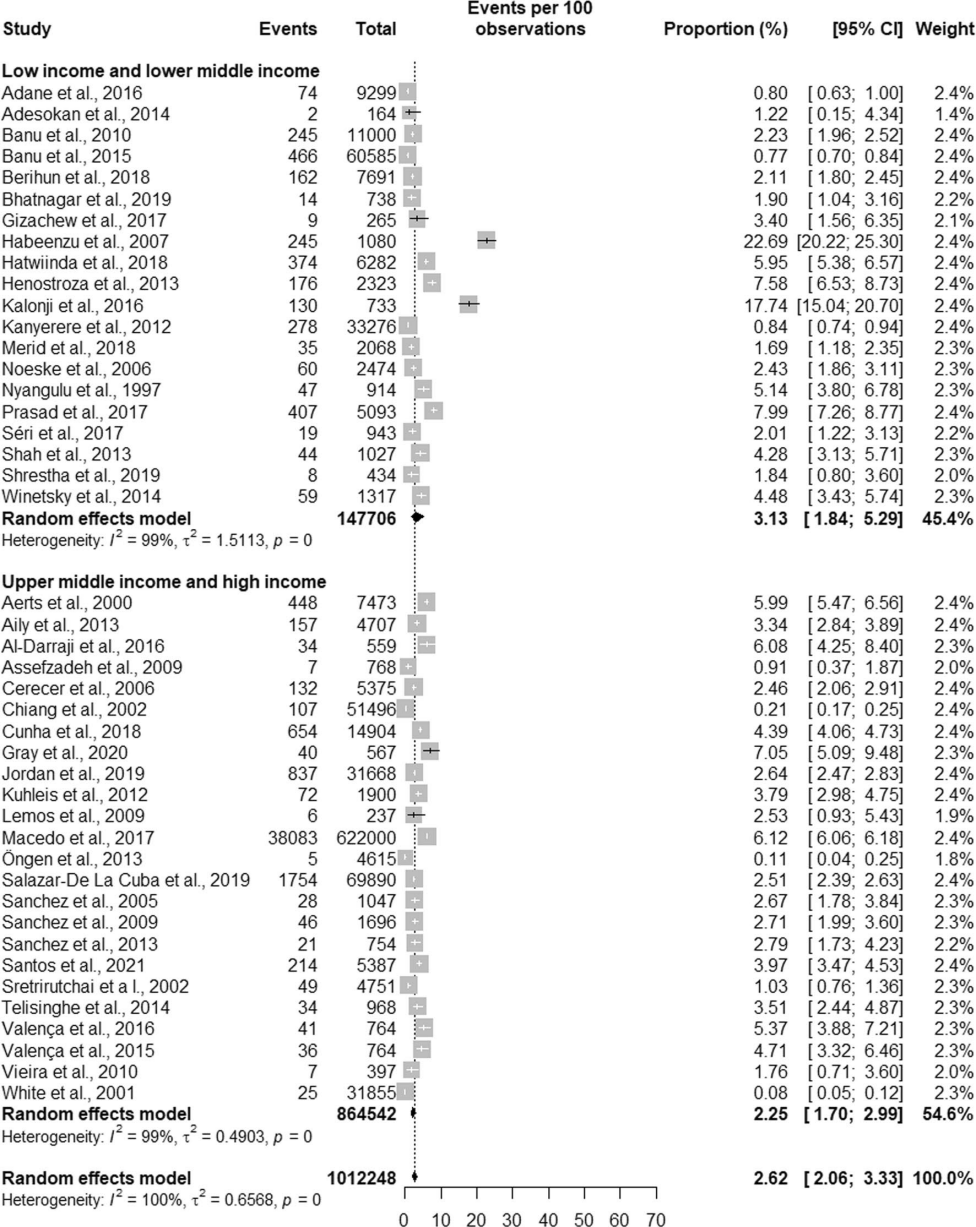
Prevalence of general tuberculosis by income level of the country

In terms of development, in low- and lower-middle-income countries, the prevalence of active TB was 3.13% [1.84; 5.29] for a total population of 147,706 prisoners. In high- and upper-middle income countries the prevalence was 2.25% [1.70; 2.99] with a population of 864,542 prisoners. Adding up all countries, the prevalence was 2.62% [2.06; 3.33] with a total of 1,012,248 prisoners (see Fig. 5).

**Fig. 6:**
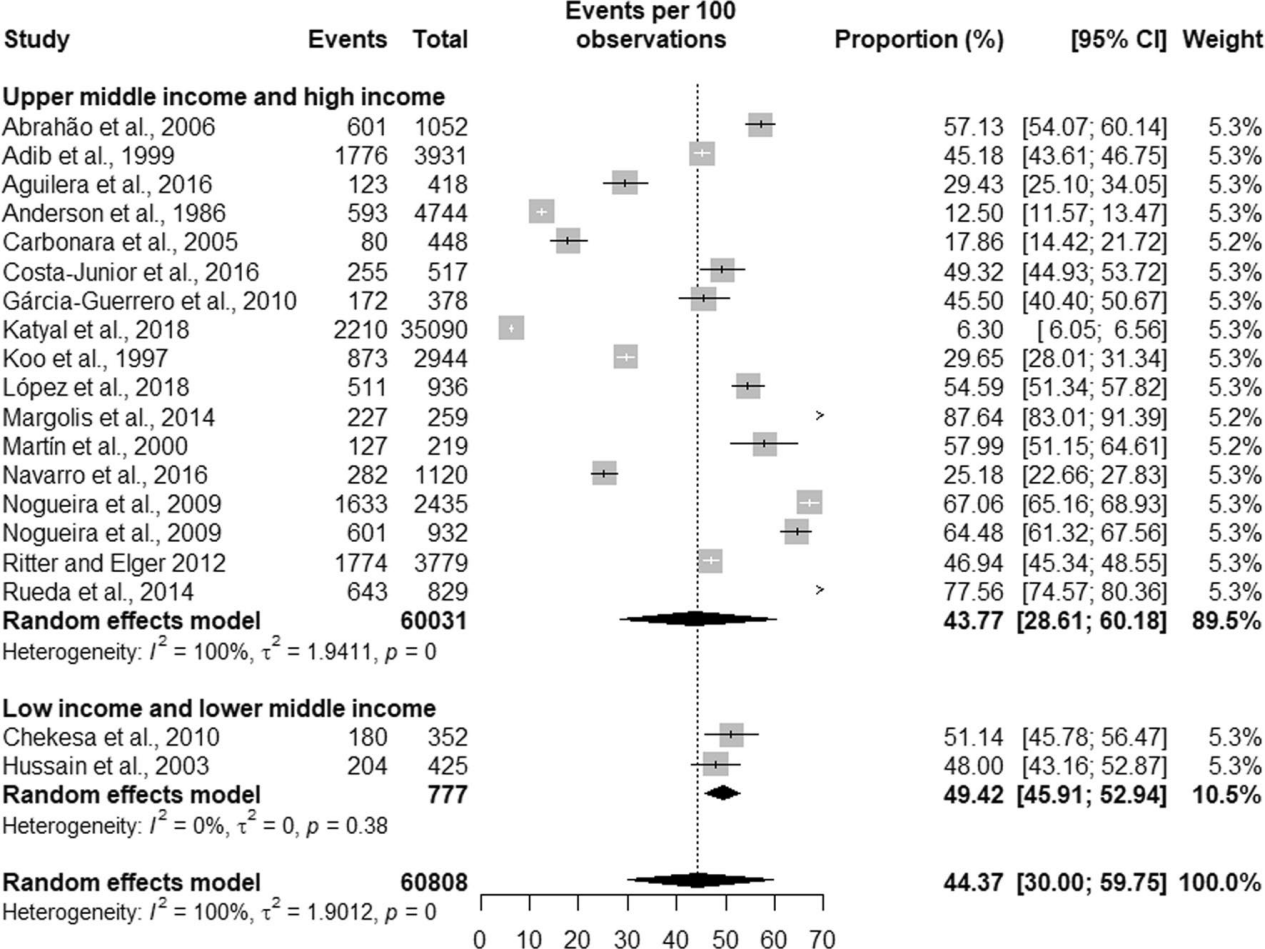
Prevalence of latent tuberculosis level of the country

The prevalence of latent TB in high- and middle-income countries was 43.77% [28.61; 60.18] in a population of 60,031 prisoners and of 49.42% [45.91; 52.94] in low and lower middle-income countries with a population of 777 prisoners. The total prevalence of latent TB was 44.37% [30.00; 59.75%] in a total of 60,808 prisoners (see Fig. 6).

**Fig. 7:**
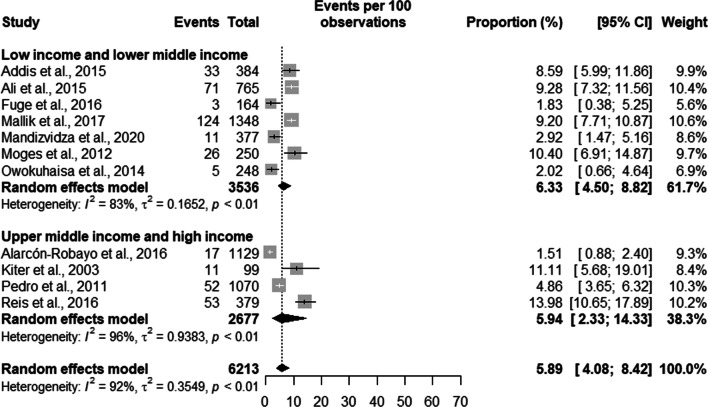
Prevalence of symptomatic tuberculosis by income level of the country

Symptomatic TB had a prevalence of 6.33% [4.50;8.82] in low- and lower-middle-income countries with a total population of 3536 prisoners, and a prevalence of 5.94% [2.33; 14.33] in a population of 2677 in high- and upper-middle-income countries. Overall, the prevalence of TB in symptomatic patients was 5.89% [4.08;8.42] in a population of 6,213 prisoners (see Fig. 7).

**Fig. 8:**
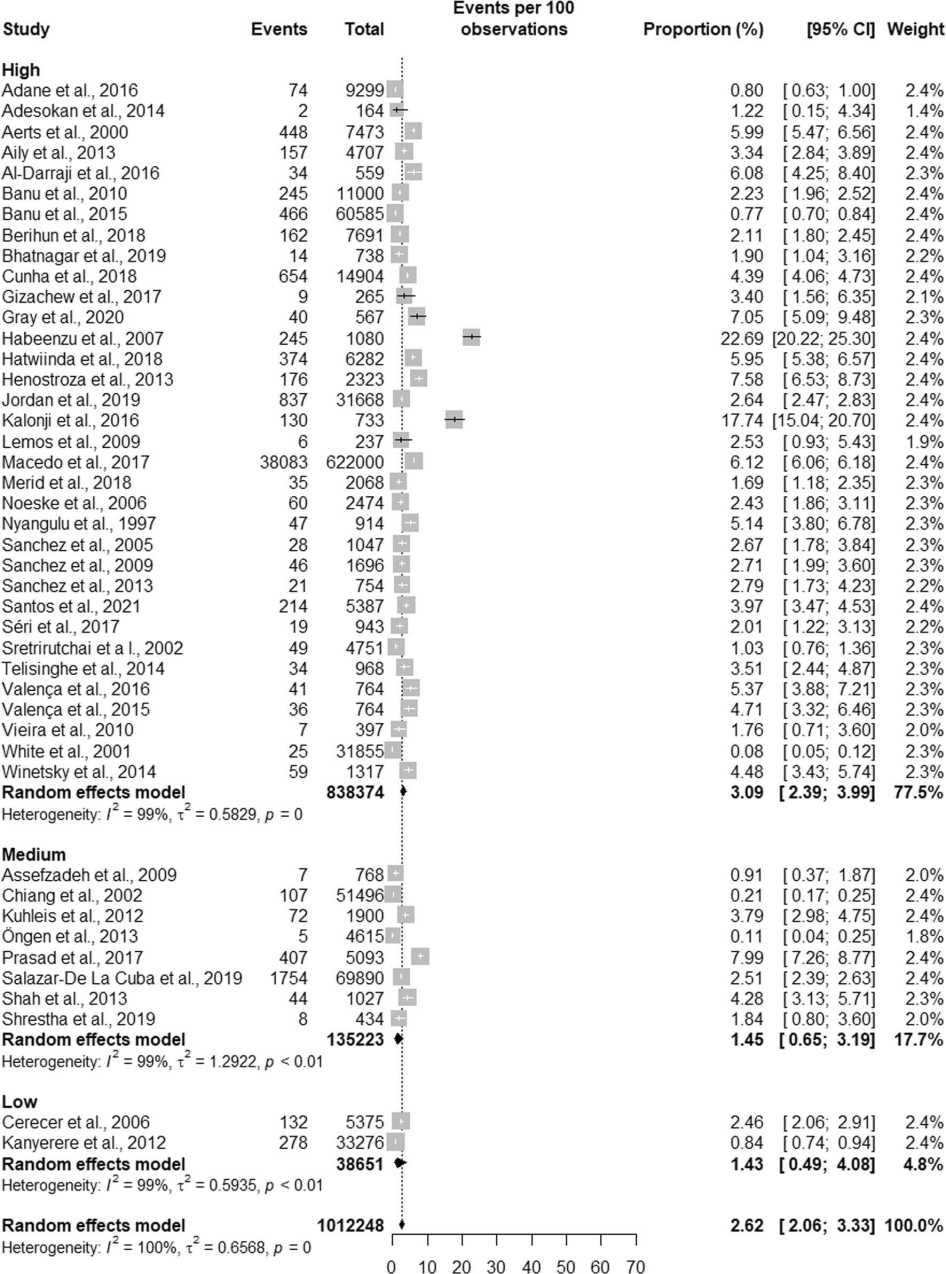
Prevalence of general tuberculosis according to the quality of the studies

Active TB had a prevalence of 3.09% [2.39;3.99] in a population of 838,374 prisoners in high-quality studies. In studies with average quality, the prevalence of TB was 1.45% [0.65; 3.19] in a population of 135,223 prisoners, and in low quality studies the prevalence of TB was 1.43% [0.49; 4.08] in a population of 38,651 prisoners (see Fig. 8).

**Fig. 9:**
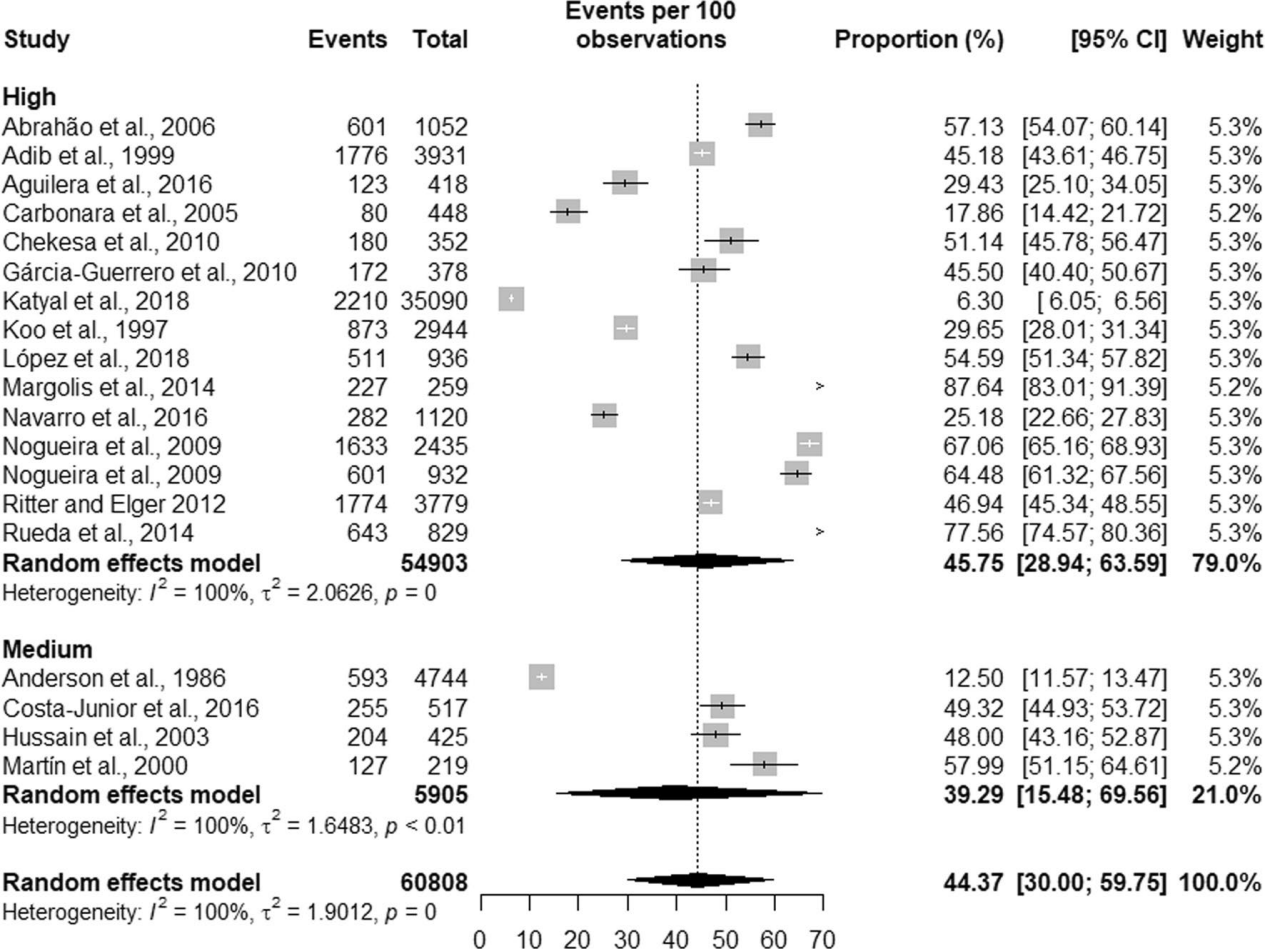
Prevalence of latent tuberculosis according to the quality of the studies

Latent TB had a prevalence of 45.75% [28.94; 63.59] in a population of 54,903 prisoners in high-quality studies. In studies with average quality, the prevalence was 39.29% [15.48; 69.56] in a population of 5,905 prisoners (see Fig. 9).

**Fig. 10:**
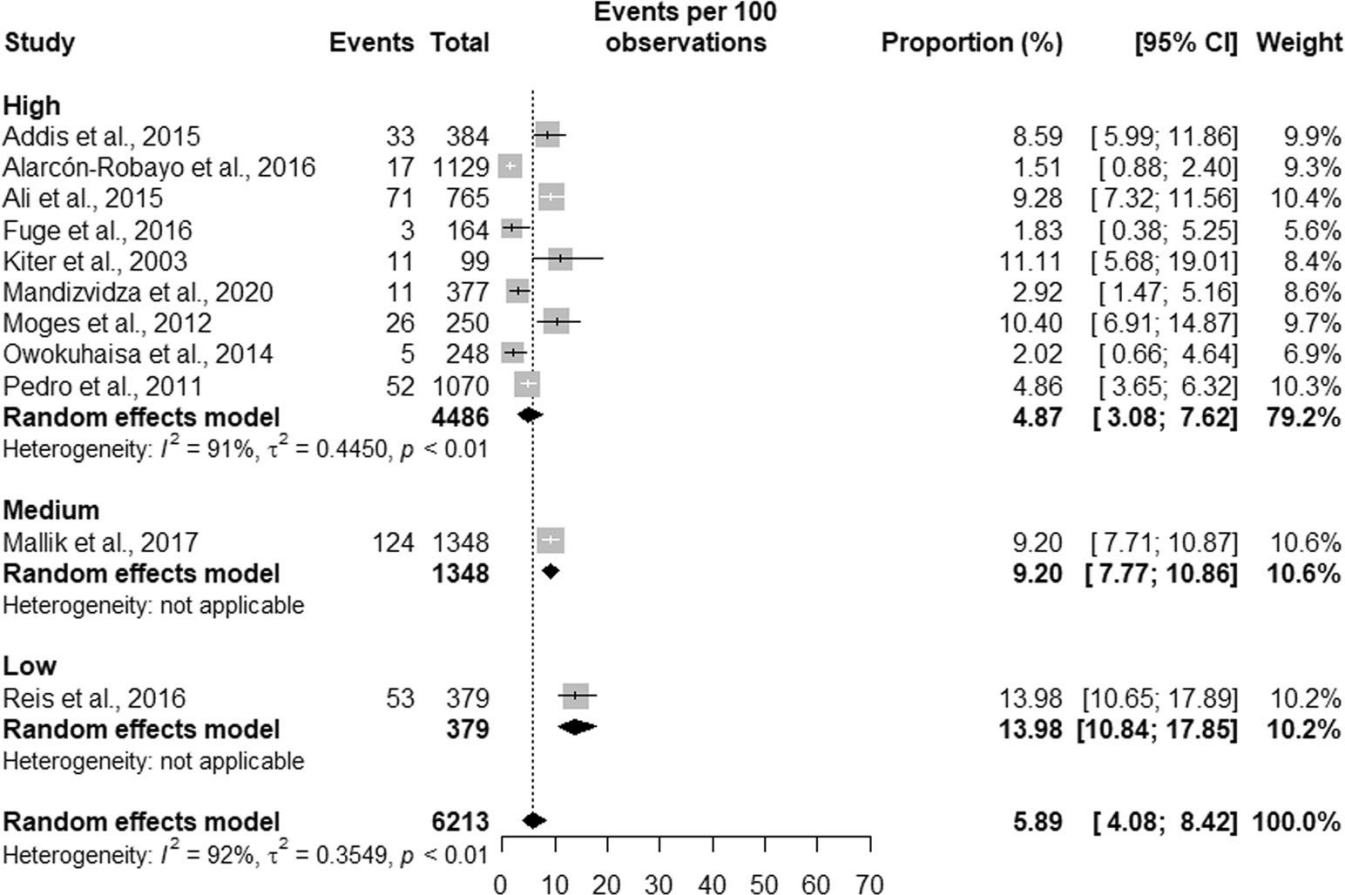
Prevalence of Symptomatic tuberculosis according to the quality of the studies

TB in symptomatic patients had a prevalence of 4.87% [3.08; 7.62] in a population of 4486 prisoners in high-quality studies. In studies with average quality, the prevalence was 9.20% [7.77; 10.86] in a population of 1,348 prisoners. In low quality studies the prevalence of TB in symptomatic patients of 13.98% [10.84; 17.85] in a population of 379 prisoners (see Fig. 10).

**Fig. 11:**
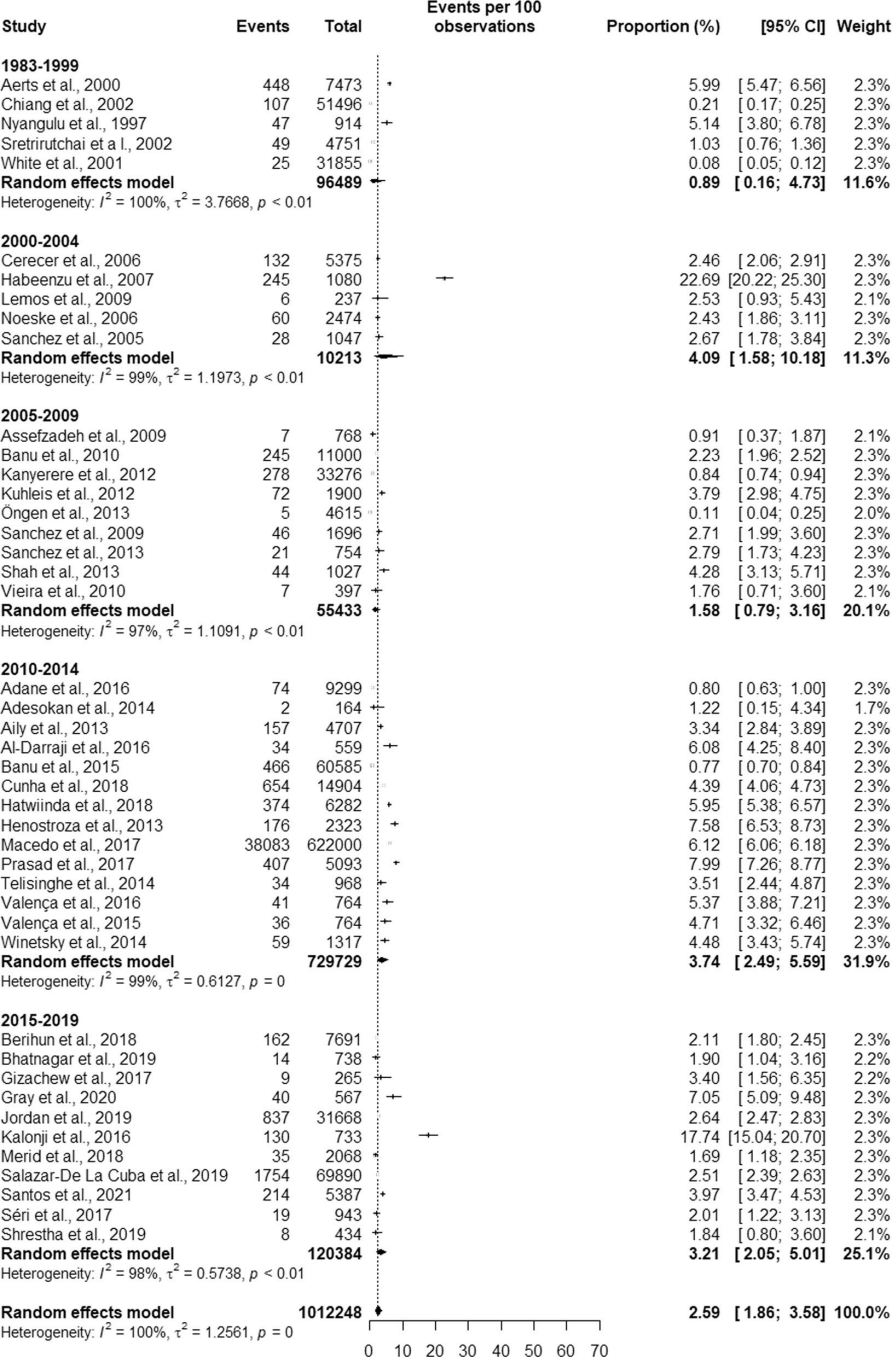
Trend in the prevalence of tuberculosis in prisoners

In the analysis of the trend of TB prevalence in the studies, we found a TB prevalence of 0.89% [0.16; 4.73] for studies from the 80 s and 90 s. Studies between the years 2000 and 2004 had a prevalence of 4.09% [1.58; 10.18], between 2005 and 2009 the prevalence was 1.58% [0.79; 3.16], between 2010 and 2014 the prevalence was 3.74% [2.49; 5.59] and the studies between 2015 and 2019 resulted in a prevalence of 3.21% [2.05; 5.01]. The total prevalence of TB in the studies was 2.59% [1.86; 3.58] (see Fig. 11).

**Fig. 12:**
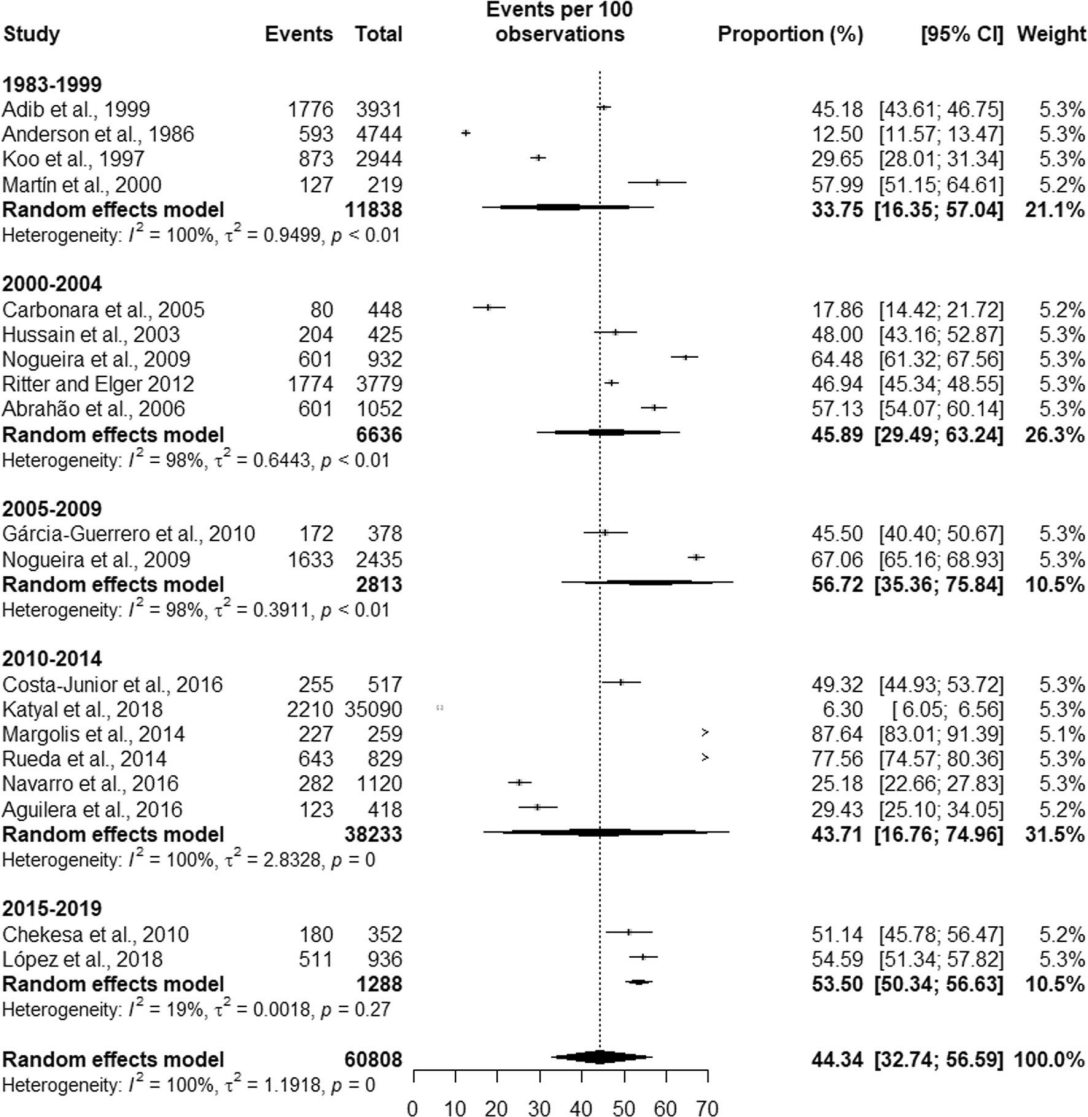
Trend in the prevalence of latent tuberculosis in prisoners

Trend analysis of latent TB prevalence was 33.75% [16.35; 57.04] in studies from the 1980s and 1990s, a prevalence of 45.89% [29.49; 63.24] between 2000 and 2004, the prevalence between 2005 and 2009 was 56.72% [35.36; 75.84], we found a prevalence of 43.71% [16.76; 74.96] between 2010 and 2014 and a prevalence of 53.50% [50.34; 56.63] between 2015 and 2019. The total prevalence of latent TB in the studies was 44.34% [32.74; 56.59] (see Fig. 12).

**Fig. 13:**
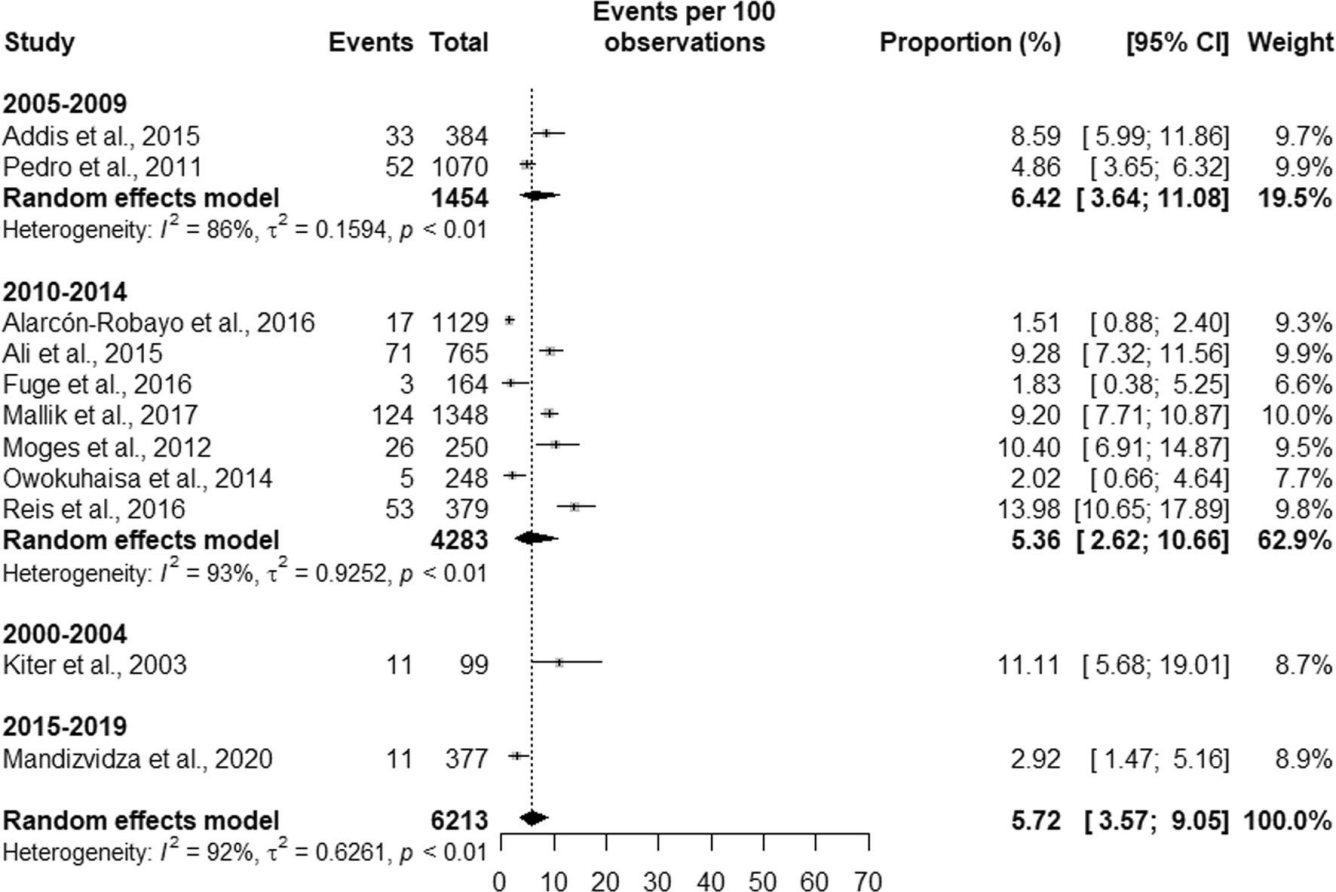
Trends in the prevalence of tuberculosis in symptomatic prisoners

The trend analysis of the prevalence of TB in symptomatic patients was 6.42% [3.64; 11.08] between the years 2005 and 2009, between 2010 and 2014 the prevalence was 5.36% [2.62; 10.66]. Between the years 2000 and 2004 and between 2015 and 2019 we found only one study in each period with a prevalence of 11.11% [5.68; 19.01] and 2.92% [1.47; 5.16]. The overall prevalence of TB in symptomatic patients was 5.72% [3.57%; 9.05] (see Fig. 13).

## Discussion

This paper reports the results on a metanalysis of the prevalence of TB, latent TB in prisoners and TB in prisoners presenting suggestive symptoms of TB reported by studies included on a broader scoping review addressing the epidemiology of TB in this population.

A total of 74 papers reported data on prevalence of TB. The combined analysis of data revealed that the prevalence of active TB was 2.5 times higher in high TB burden countries when compared to low burden countries [1]. The latent TB prevalence was also higher (1.2 times) in high TB burden countries. The same trend was seen in studies assessing the prevalence of TB in prisoners with suggestive symptoms of TB, where the prevalence of the disease was 2.3 times higher in countries with high TB burden.

These findings were not a novelty since it would be expected for prisoners in countries with high burden of TB to also have higher prevalence of TB (active or latent). Prisons produce favourable conditions for the expansion of the TB epidemic in the general population. A study carried out in Brazil showed that incarcerated individuals enter prisons with a low risk of TB [101]. However, this risk increases rapidly over 5 years, reaching a peak of more than 1300 cases per 100,000 person-years, being 30 times greater than that of the general population. When prisoners are released and return to the community, they present a 5.5 times greater risk of active TB than the general population. The risk remains elevated for 7 years [101].

When comparing studies conducted in low- and low-middle-income countries with those in high and high-middle income, it was also possible to observe higher prevalence in low- and low-middle-income countries of active and latent TB and among symptomatic prisoners [25]. However, there were only two studies on latent TB in low- and middle-low-income countries, with a small number of participants, reason why results should be carefully interpreted.

In this analysis, only 22 studies were conducted in high burden TB countries who were middle/high income countries (Brazil, Thailand and South Africa), with 19 of them being conducted in Brazil. The remaining studies were done in low- and lower-middle-income countries with high burden of disease. More than 90% of reported TB infections occur in low- and middle-income countries [102]. Low-income contexts tend to be more prone to racial issues inside and outside prisons. Brown and black populations suffer more individual and social deprivation in different parts of the world, making their surroundings deficient, exhausting and a potential disease generator. Inside prisons, this situation is no different, studies show that in this environment presents greater notification of TB in brown and black groups and with low education [103–105]. In addition, the prison population in general also belongs to this social group, as in Brazil, 67% of its prison population is black or mixed-race and 61% are illiterate or with low education [106, 107].

High quality studies had active TB prevalence rates *circa* 2.1 times higher than medium and low-quality studies. In relation to latent tuberculosis, high-quality studies had higher rates than those of medium quality. In studies that included symptoms of TB, the rates were higher in the two medium and low quality ones, thus resulting in a frail evidence of this difference.

The global prevalence of latent TB in the general population according to the World Health Organization-WHO is a quarter of the world's population [108]. A systematic review with meta-analysis was performed in 2019 revealed a prevalence of 24.8% (95% CI 19.7–30.0%) of latent TB in the general population worldwide [109]. These data show a discrepancy in relation to the data found in our study with the prisoner population.

In the general population, in 2018, about 10 million people were notified with TB worldwide, equivalent to 132 per 100,000 population a 2% decline from 2017. From 2000 to 2018 there was a 1.6% drop in the incidence of TB in the general population [110]. According to the WHO, over a 20-year period, the overall incidence of TB ranged from a maximum of 174 cases per 100,000 population in the year 2000 to a minimum of 127 cases per 100,000 population in the year 2020, these numbers are lower than those found in the prisoner population [111].

Focusing interventions on prisons can have powerful effects in reducing TB rates in the general population as investing in tuberculosis control in 0.5% of the population would reduce the total incidence of active TB in the population by more than 40% [101].

## Conclusion

Prisons are potentially transmitting place for TB: not only due to its poor structure conditions but also because of the social issues that migrate from the outside world with the imprisonment of individuals.

The metanalysis conducted suggests that TB, and probably other infectious diseases, find fertile ground in prisons where previous acquired social disadvantages seem to thrive.

Therefore, TB in prisons should be understood as a global public health problem because TB is not only a health problem, but also a social problem, effective strategies to control the disease are needed targeting the prison environment, including rapid health assessments to understand each context and to implement tailored and precision interventions.

## Supplementary Information

**Additional file 1.** Search strategy.

## Data Availability

All data generated or analyzed during this study are included in this publication. The references of the analyzed articles are available in Table 2 of this manuscript. Additional file 1 includes the sequence of words and databases used for the search.

## Abbreviations

TB: Tuberculosis
HIV: Human Immunodeficiency Virus
AIDS: Acquired Immunodeficiency Syndrome
SR: Scoping review
MDR-TB: Multidrug-resistant tuberculosis
WHO: World Health Organization
