# Supplementary material for: Epidemiology of TB in prisoners: a metanalysis of the prevalence of active and latent TB
Source: BMC Infect Dis. 2023 Jan 11;23:20. doi: 10.1186/s12879-022-07961-8 (PMC9835258; doi:10.1186/s12879-022-07961-8)
Supplement: Supplementary file 1 — Additional file 1. Search strategy. [file 12879_2022_7961_MOESM1_ESM.docx]

**Search Strategy**

**PUBMED:** (Tuberculosis OR Kochs Disease OR Koch's Disease OR Koch Disease OR Mycobacterium tuberculosis Infection OR Infection, Mycobacterium tuberculosis OR Infections, Mycobacterium tuberculosis OR Mycobacterium tuberculosis Infections) **AND** (Prisons OR Prison OR Penitentiaries OR Penitentiary OR Prisoners OR Prisoner OR Prison inmate)

**LILACS:** (Tuberculosis OR Kochs Disease OR Koch's Disease OR Koch Disease OR Mycobacterium tuberculosis Infection OR Infection, Mycobacterium tuberculosis OR Infections, Mycobacterium tuberculosis OR Mycobacterium tuberculosis Infections) (WORDS)

AND (Prisons OR Prison OR Penitentiaries OR Penitentiary OR Prisoners OR Prisoner OR Prison inmate) (WORDS)

**SCIELO:** (tuberculosis) AND (prisons) OR (prison) AND (prisoners) OR (prisoner) OR (prison inmate)

# SCOPUS: (Tuberculosis OR "Kochs Disease" OR "Koch's Disease" OR "Koch Disease" OR "Mycobacterium tuberculosis Infection" OR "Mycobacterium tuberculosis Infections")

# AND (Prisons OR Prison OR Penitentiaries OR Penitentiary OR Prisoners OR Prisoner OR "Prison inmate")
